# Evaluation of Cardiorespiratory Function During Cardiopulmonary Exercise Testing in Untreated Hypertensive and Healthy Subjects

**DOI:** 10.3389/fphys.2018.01590

**Published:** 2018-11-14

**Authors:** Yahui Zhang, Zhihao Jiang, Lin Qi, Lisheng Xu, Xingguo Sun, Xinmei Chu, Yanling Liu, Tianjing Zhang, Stephen E. Greenwald

**Affiliations:** ^1^Sino-Dutch Biomedical and Information Engineering School, Northeastern University, Shenyang City, China; ^2^Key Laboratory of Cardiovascular Disease, Fuwai Hospital, National Center for Cardiovascular Disease, Chinese Academy of Medical Science, Beijing, China; ^3^Beijing Haidian Hospital, Peking University Third Hospital Haidian Campus, Beijing, China; ^4^Beijing Rehabilitation Hospital of Capital Medical University, Beijing, China; ^5^Blizard Institute, Barts, The London School of Medicine, Dentistry, Queen Mary University of London, London, United Kingdom

**Keywords:** SDPPG, cardiorespiratory function, untreated hypertensive subject, healthy subject, cardiopulmonary exercise testing (CPET)

## Abstract

**Objective:** This study aimed to compare differences in cardiorespiratory function between untreated hypertensive subjects (UHS) and healthy subjects (HS) during cardiopulmonary exercise testing (CPET). Additionally, it also aimed to explore the potential mechanisms of different exercise responses in cardiorespiratory function before, during and after CPET.

**Methods:** Thirty subjects (15 UHS and 15 HS) were enrolled. Photoplethysmography (PPG), respiratory signal, and ECG were simultaneously collected while subjects were performing CPET. Fiducial points (a, b, c, d, e) were extracted from the second derivative of the PPG (SDPPG), and the ratios b/a, c/a, d/a, e/a, and (b-c-d-e)/a (named Aging Index, AGI) were calculated as markers of systolic and diastolic function. Additionally, respiratory rate was calculated and analyzed.

**Results:**Before CPET, there were no significant differences in b/a, d/a, and AGI between two groups. However, after CPET, b/a (−0.9 ± 0.19 vs. −1.06 ± 0.19, *p*-value = 0.03) and AGI (−0.49 ± 0.75 vs. −1.15 ± 0.59, *p*-value = 0.011) of the UHS group were significantly higher than those of the HS. The d/a (−0.32 ± 0.24 vs. −0.14 ± 0.17, *p*-value = 0.024), and c/a (−0.33 ± 0.26 vs. −0.07 ± 0.19, *p*-value = 0.004) were significantly lower in UHS than those in HS. In contrast, before CPET, e/a (0.22 ± 0.11 vs. 0.32 ± 0.09, *p*-value = 0.007) in UHS was significantly lower than that in HS, while after CPET there was no significant difference between the two groups in this variable. In addition, during CPET, AGI (*p*-value = 0.003), and respiratory rate (*p*-value = 0.000) in UHS were significantly higher in comparison with before CPET.

**Conclusions:** Different exercise responses showed the differences of cardiorespiratory function between UHS and HS. These differences not only can highlight the CV risk of UHS, but also can predict the appearance of arterial stiffness in UHS. Additionally, during CPET, significant differences in AGI, autonomic nervous function and respiratory activity assessed by respiratory rate were found between the two groups in comparison with before CPET.

## 1. Introduction

Hypertension is the most common cause of, and a major predisposing risk factor for, cardiovascular morbidity and mortality. It has been associated with the progression of atherosclerosis and cerebrovascular disease (Dominguez-Rodriguez et al., 2013; Nikolic et al., 2014). While the development of cardiovascular disease (CVD) is associated with changes in the structure and function of arteries, it is accelerated by the presence of various risk factors, one of the most important of which is hypertension (Webb, 2000). Based on its multi-factorial and complex nature, the underlying causes of hypertension need to be further identified. Photoplethysmography (PPG), a simple, non-invasive optical measurement technique can be used to monitor the change of blood volume in the microvascular beds of peripheral tissues (Allen, 2007; Chang et al., 2016). The second derivative of the PPG signal (SDPPG) has been widely used as a simple and reliable measure of pressure augmentation in clinical investigations (Hashimoto et al., 2002). It not only reflects the ascending aortic systolic pressure wave, but also serves as a marker of vascular aging (Takazawa et al., 1998; Hashimoto et al., 2002; Elgendi, 2012). In addition, it is also closely associated with cardiovascular risk indicators (Otsuka et al., 2007; Yousef et al., 2012; Laukkanen and Rauramaa, 2013). Fiducial points (a, b, c, d, e) were extracted from the SDPPG, and the ratios b/a, c/a, d/a, e/a and (b-c-d-e)/a were calculated as markers of systolic and diastolic function. We note here that these five fiducial points and the quantities derived from them, which in the last 20 years have become widely used (Takazawa et al., 1998), are purely an empirical description of the SDPPG. We emphasize that there is no clear theoretical or experimental description of the link between these quantities and the underlying mechanical properties of the cardiovascular system. Nevertheless, the derived quantities reflect aspects of arterial stiffness and wave propagation and have been related to aging and various cardiovascular pathologies. Thus, they provide a straightforward way of quantifying PPG signals and deriving useful prognostic and diagnostic information from them.

Some studies have investigated SDPPG indices in patients with hypertension, however, these results remain controversial, and the mechanism was still unclear. For instance, Hashimoto et al. (2005) and Kaibe et al. (2002) found that SDPPG can be useful for the assessment of vascular aging or for detection of arteriosclerotic disease. It is useful for SDPPG to non-invasively measure the vascular aging accelerated by hypertension, even in untreated hypertensive patients (Hashimoto et al., 2005). A study found that d/a was significantly related to the risk of metabolic syndrome and hypertension (Tomoyuki and Toshiaki, 2013). In contrast, there was no relationship between the SDPPG and inflammation in middle-aged men with various cardiovascular risks, including hypertension (Otsuka et al., 2007). Moreover, it has been shown that, although the SDPPG indices reflect changes in arterial structure and function, they have limited diagnostic value for pathophysiological changes both in large arteries and small vessel disease (Tabara et al., 2016).

In addition, some studies have reported a link between cardiovascular and respiratory function (Martinez et al., 2011). Hjortdal et al. found that inspiration promoted inferior vena cava flow at rest. Meanwhile, venous return in the total cavopulmonary connection circulation is affected by cardiac output, respiration and the peripheral venous pump (Hjortdal et al., 2003). Barthel et al. showed that respiratory rate predicted outcome following of acute myocardial infraction. However, the precise mechanisms of this link still need to be further disclosed (Barthel et al., 2013).

Furthermore, cardiopulmonary exercise testing (CPET) is regarded as the gold standard for the comprehensive assessment of exercise response (Wibmer et al., 2014) and diagnosis of cardiopulmonary functional capacity (Dominguez-Rodriguez et al., 2013). Some studies have reported that CPET can predict and evaluate cardiovascular risk (Dominguez-Rodriguez et al., 2013; Laukkanen and Rauramaa, 2013). However, there have been very few studies investigating cardiorespiratory function in response to exercise in CVD by SDPPG analysis. For example, exercise causes a significant increase in heart rate, respiratory rate, muscle sympathetic nerve activity and skin blood flow (Brown et al., 2013). In addition, inspiration facilitated vascular function at rest but less so during exercise in patients (Hjortdal et al., 2003). However, the link between the response to exercise and these changes in cardiorespiratory function remains unclear.

Therefore, this study aimed to investigate and compare cardiorespiratory function in untreated hypertensive and healthy subjects before, during and after CPET. Additionally, it aimed to better understand the possible causes for the aforementioned lack of consensus based on the measurement and calculation of synchronous multi-physiological signals, including PPG, ECG, and respiratory signal.

## 2. Methods

### 2.1. Subjects

Thirty subjects [15 untreated hypertensive subjects (UHS) and 15 HS] were enrolled from the Fuwai Hospital, National Research Center of Clinical Medicine for Cardiovascular Diseases, Beijing Rehabilitation Hospital and Beijing Haidian Hospital. The basic characteristics of subjects in this study are shown in Table 1. Inclusion criteria were as follows: (1) only primary hypertension (SBP: 140–159 mmHg or DBP: 90–99 mmHg) and not taking anti-hypertensive medication; (2) no other CVD; (3) sedentary lifestyle; (4) smoking or drinking (less than once a month). All subjects gave informed consent, and the protocol was approved by the ethics committee of the Fuwai Hospital, Beijing Rehabilitation Hospital, and Beijing Haidian Hospital.

**Table 1:** Characteristics of the study subjects.

	**Untreated hypertension subjects**	**Healthy subjects**	***p*-value**
No.	15	15	–
Male, gender, [*n*(%)]	10 (67)	7 (47)	0.072
Age [*year*]	48 ± 11.5	44 ± 11.8	0.355
Height [*cm*]	169.8 ± 6.1	163.3 ± 5.9	0.006
Weight [*kg*]	78.6 ± 9.7	64.8 ± 8.6	0.003
BMI [*kg*/*m*^2^]	27.3 ± 3.5	24.3 ± 3.07	0.018
SBP [*mmHg*]	142.8 ± 7.5	112.8 ± 6.5	0.006
DBP [*mmHg*]	90.2 ± 11.0	78.2 ± 11.3	0.517
HR [*bpm*]	73.3 ± 10.1	62.8 ± 7.8	0.945
Smoking history [*n*(%)]	9 (60)	4 (26.7)	0.069
Drinking [*n*(%)]	8 (53.3)	5 (33.3)	0.285

*Data are mean ± SD; SBP, systolic blood pressure; DBP, diastolic blood pressure; HR, heart rate*.

### 2.2. Study protocol

The experiment was conducted at the key laboratory for CPET with temperature maintained between 20 and 22°C. All the subjects underwent a CPET (COSMED S. R. L.) on a cycle ergometer (COSMED) using an incremental protocol consisting of the following steps: 3-min rest on the cycle ergometer, 5-min no-load warm up, 6–10-min incremental exercise test, and 5–10-min recovery. The incremental rate was 20–30 W/min until individual exhaustion. The PPG, respiratory signal, and electrocardiogram (ECG) were continuously recorded with the SOMNOscreen® polysomnography device (SOMNOmedics GmbH, Randersacker, Germany). The sampling rate of PPG, respiratory signal and ECG was 128, 32, and 256 Hz, respectively. PPG signal was recorded by plethysmographic curve of pulse oximetry of the finger, and respiratory signal was recorded by Rip-type thoracic and abdominal motion sensor. These signals were simultaneously collected before, during and after CPET in two groups.

### 2.3. Data processing

**Figure 1:**
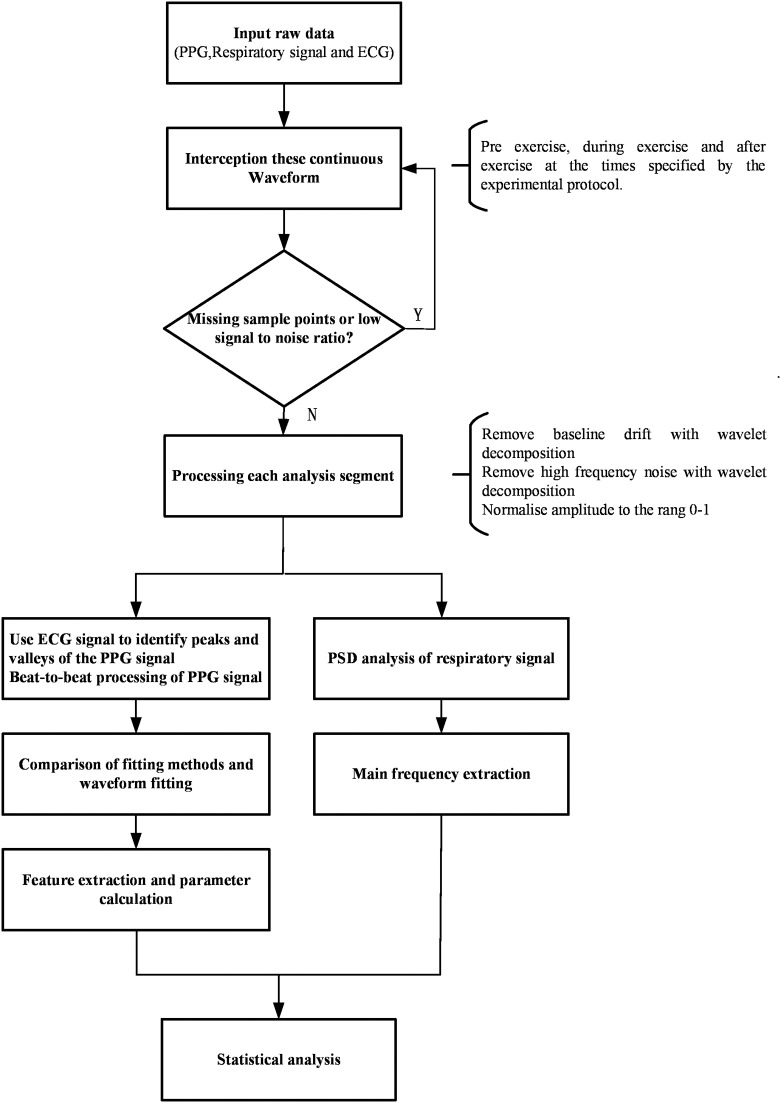
Diagram of signal processing procedure.

**Figure 2:**
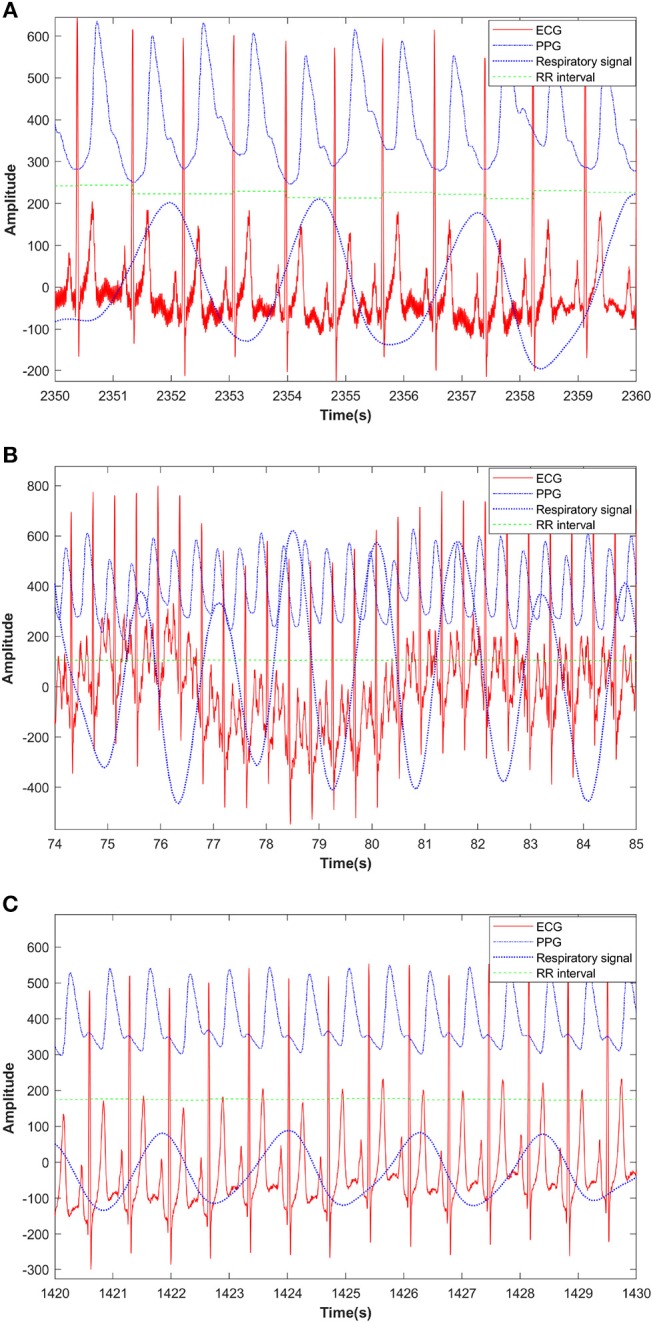
PPG, respiratory signal, ECG and RR interval from a pre-exercise segment **(A)**, during exercise segment **(B)**, and after exercise segment **(C)** from a healthy subject.

**Figure 3:**
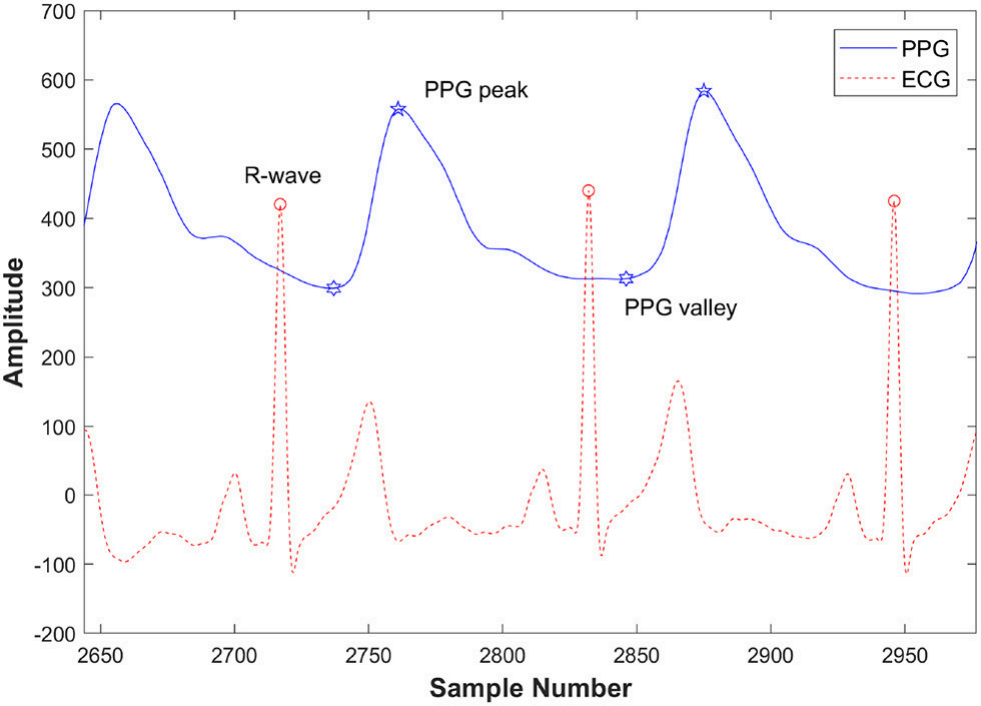
Detection of peaks and valleys of the PPG signal with the ECG R-wave in a pre-exercise segment from a healthy subject.

The data processing analysis is shown in the diagram (Figure 1). The raw data (PPG, respiratory signal, and ECG) were intercepted and recorded by segments (before, during, and after CPET) thus separating the lower quality data recorded during CPET, including missing sample points and low signal-to-noise ratio. These segments were selected according to the time points of this study protocol which includes rest, incremental exercise and recovery phases. The 20–40 continuous and high-quality waveforms were selected in each segment. The raw data (PPG, respiratory signal, and ECG) and RR interval from the pre-exercise segment, during and after-exercise from a healthy subject are illustrated in Figure 2. PPG of each recorded segment was pre-processed by removal of baseline drift and de-noising. Removal of baseline drift and de-noising were used by the wavelet basis of db7. Third-layer and seventh-layer wavelet decomposition were used to remove the high-frequency noise and to get low-frequency baseline, respectively. The beat-to-beat PPG was segmented based on each trough point, and then the amplitude in Y-axis was normalized to 1. Next, the peaks and valleys of the signal during each cardiac cycle were found by using the R-wave of the ECG as a fiducial marker (Figure 3), having first identified the R wave from the 1st derivative of the ECG signal. A sliding window approach was used to get the point among R-wave and S-wave which is the minimum slope of the ECG signal in every cardiac cycle, given that R-peak occurred before the ECG maximum slope.

### 2.4. Second derivative curve fitting

**Figure 4:**
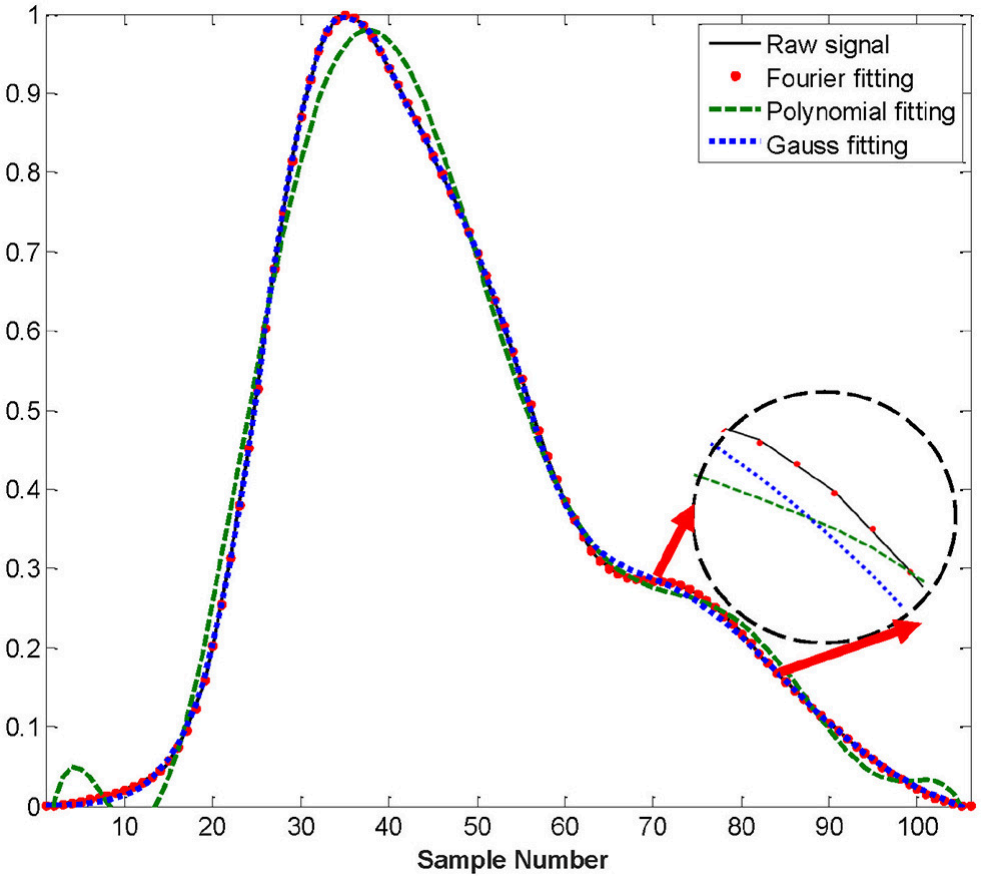
Difference waveform fittings of PPG in a cardiac cycle before exercise from a healthy subject.

The second derivative of the PPG waveform derived directly from the raw signal did not reliably detect the required key features (points a, b, c, d, and e). Therefore, we chose to adopt a fitting approach to obtain a representation of the second derivative from which the key features could be obtained analytically. We compared three common methods of waveform fitting (Fourier, polynomial, and Gaussian fitting) in order to get a more accurate fit. These fitting methods of PPG in a cardiac cycle before exercise from a healthy subject are illustrated in Figure 4. According to Figure 4, Fourier fitting was more accurate than other methods. The root mean square error (RMSE) was calculated and compared among these methods before, during and after exercise. Before exercise, the RMSE of Fourier, polynomial and Gaussian fitting was 0.0014, 0.029, and 0.005, respectively. The results also showed that the Fourier fitting provided a better fit to this waveform during exercise (RMSE = 0.00023) and after exercise (RMSE = 0.0035) in comparison with other methods. These results of RMSE were listed in Table 2. Therefore, the SDPPG was obtained from Fourier fitting function. The Fourier fitting was computed by Proakis and Manolakis (1996):

(1) $$F(x)=a_{0}+\sum_{i=1}^{n}\left(a_{i}\cdot cos(iwx)+b_{i}\cdot sin(iwx)\right)+e,$$

where *n* is the model order, and *a*_*i*_ and *b*_*i*_ are the fitting parameters. *e* is noise or fitting error.

**Table 2:** These results of RMSE in different fitting methods.

	**Before exercise**	**During exercise**	**After exercise**
Fourier	0.0014	0.00023	0.0035
Polynomial	0.029	0.0051	0.022
Gaussian	0.005	0.0022	0.0094

In this study, the Fourier model was characterized by 18 parameters: Θ = {ω,*a*_0_,*a*_*i*_,*b*_*i*_, i = 1,2,3,…,8}. These parameters can be determined by solving the following optimization problem, which can be achieved by nonlinear least square fitting:

(2) $$\Theta =\underset{\theta \in D}{\arg\;\min}\|f(x)-F(x){\|}_{2}$$

where *f*(*x*) is the input waveform of each cardiac cycle, and *D* is the parameter space.

**Figure 5:**
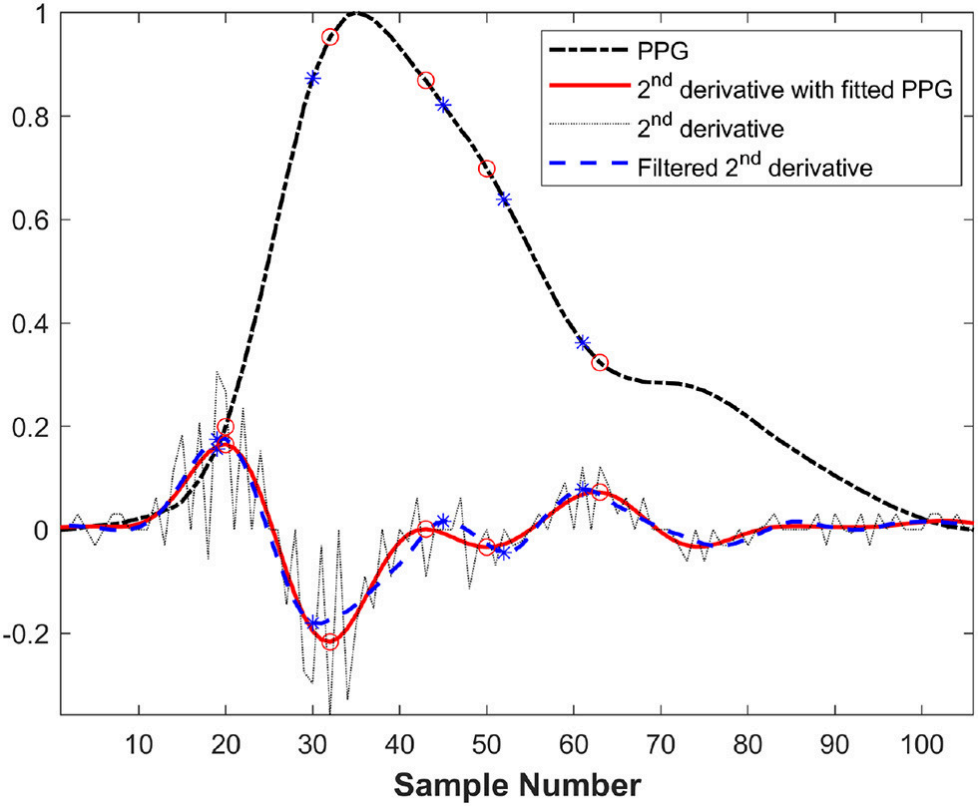
Comparison between direct second derivative and second derivative of Fourier fitting function in a cardiac cycle before exercise from a healthy subject (* and ◦ was the feature points of filtered second derivative and fitted second derivative, respectively).

The direct second derivative and Fourier fitting of PPG in a cardiac cycle are illustrated in Figure 5. In this figure, the second derivatives of two methods are magnified 20 times in order to clearly compare the effectiveness of two methods. In this figure, 2nd derivative with fitted PPG were more accurate than filtered 2nd derivative because filtering may lead to the dislocation of the feature points.

### 2.5. Feature extraction of PPG

**Figure 6:**
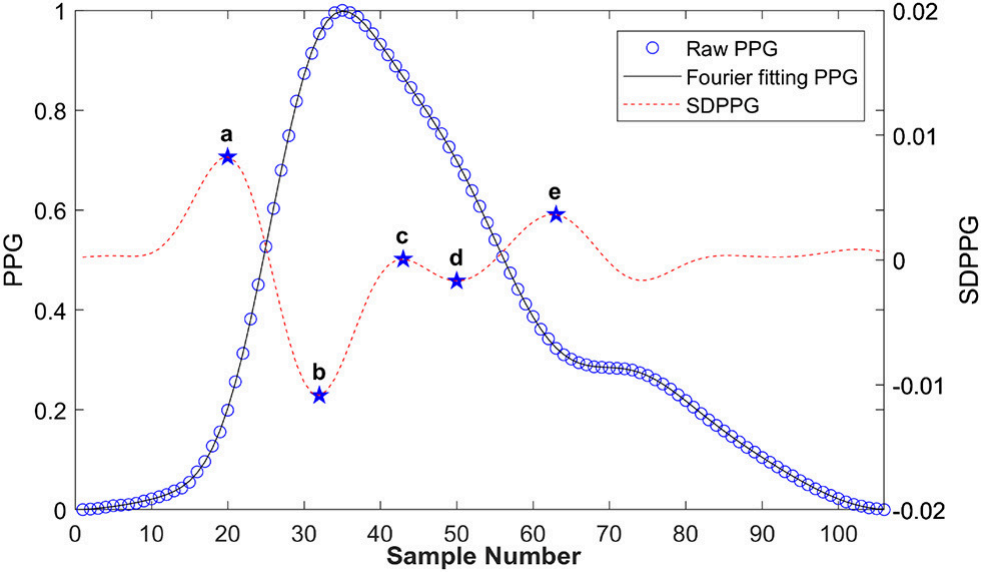
Typical PPG and its second derivative in a cardiac cycle based on Fourier fitting from a healthy subject at rest. The black solid line is the raw PPG, and the open circles are the Fourier fitted data (root mean square error (RMSE) = 0.0014, *R*^2^ = 0.99).

**Figure 7:**
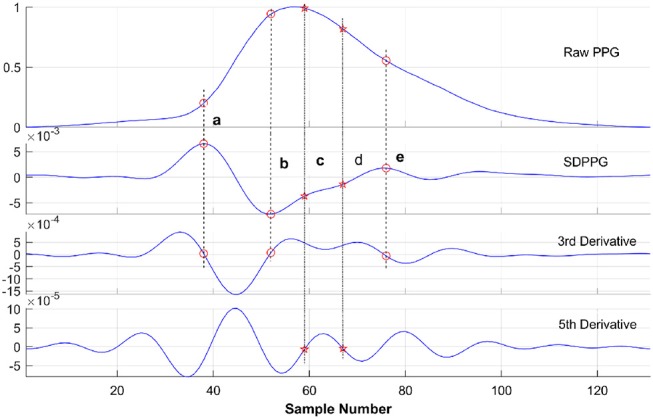
Acquisition of feature points (a, b, c, d, and e) of SDPPG in a cardiac cycle from an untreated hypertensive subject. As explained in the text most of the points were derived from the 3rd derivative. If no clear zero crossing was found (e.g., points c and d), the 5th derivative was obtained from which the zero crossings could be identified.

Figure 6 shows a typical recording of a single cardiac cycle and its second derivative based on Fourier fitting function, in a healthy subject at rest. The RMSE and *R*^2^ were 0.0014 and 0.99, respectively. The SDPPG consists of five features as follows: point a, the first peak of the cycle corresponding closely to the start of the systolic wave of the original PPG signal; point b, the following trough, associated with the systolic maximum of the PPG signal; points c, d, and e are associated with changes in the slope of the diastolic wave. Most of these feature points were derived from zero crossings in the 3rd derivative of the PPG signal as shown in Figure 7. However, in some traces points (such as c and d) that were not clearly defined in the 3rd derivative curve were derived from zero crossings in the 5th derivative. For example, in Figure 6, points c and d are clearly defined in the SDPPG and could be localized from the 3rd derivative, while in Figure 7, these points required the 5^*th*^ derivative to yield a clear zero crossing.

### 2.6. Power spectral density of respiratory signal

The raw respiratory signals before, during and after CPET were analyzed by Fast Fourier Transform. The respiratory rate was extracted as the fundamental frequency by power spectral density (Proakis and Manolakis, 1996). Each signal *s*(*n*) is modeled via an autoregressive model by:

(3) $$s(n)=-\sum_{p=1}^{m}a[p]\cdot s(n-p)+e(n),$$

where *e*(*n*) represents zero-mean white noise with variance $\delta_{e}^{2}$. The *a*[*p*] is the AR coefficients and *m* is the model order (*p* = 1, 2, …, *m*). *n* is the number of sample points in each cardiac cycle. When $\delta_{e}^{2}$ and autoregressive coefficients were estimated, the power spectral density of an autoregressive process is calculated by means of:

(4) $$P_{AR}(f)=\frac{\delta_{e}^{2}}{|1+\sum_{p=1}^{m}a[p]\cdot e^{-j\cdot 2\pi \cdot fpT}{|}^{2}},$$

where *T* is the sampling period. The order estimation was computed by Akaike's final prediction error (FPE) criterion (Akaike, 1969):

(5) $$FPE(m)=\sigma_{m}^{2}\cdot \frac{N+m-1}{N-m-1},$$

The *m* value corresponding to the minimum FPE-value is the order of the model, *m* ∈ [1, *N*/2). The $\sigma_{m}^{2}$ is the estimate of the prediction error power of current order *m*. *N* is the observation points.

### 2.7. Cardiorespiratory parameters

Based on the raw PPG, respiratory signal and ECG, we comprehensively calculated and analyzed the following cardiorespiratory variables: b/a, (i.e., the ratio of the height of the b wave to that of the SDPPG); and analogously, e/a; c/a; d/a; (b-c-d-e)/a (the so called AGI) (Takazawa et al., 1998); (b-e)/a (i.e., the disappearance of c and d during exercise); and finally, the respiratory rate, i.e., the fundamental frequency of power spectral density of respiratory signal.

### 2.8. Statistical analysis

All the data were presented as mean ± SD. The Kolmogorov–Smirnov test was conducted to check for normal distribution of the measured and calculated variables. A factorial ANOVA was used to compare differences between two groups after performing a CPET. The repeated ANCOVA was used to conduct the differences at pre-exercise, during exercise and after exercise within a group. Statistical analyses were performed with SPSS version 20.0 (IBM SPSS Statistics, USA). Significance was considered *p* < 0.05.

## 3. Results

### 3.1. SDPPG indices in UHS and HS before, during and after CPET

Continuous and high-quality 20–40 SDPPG indices were extracted from beat to beat PPG and calculated for every subject in each segment (before, during or after CPET). The SDPPG indices for each group were averaged and then tested for statistically significant differences between the two groups.

**Figure 8:**
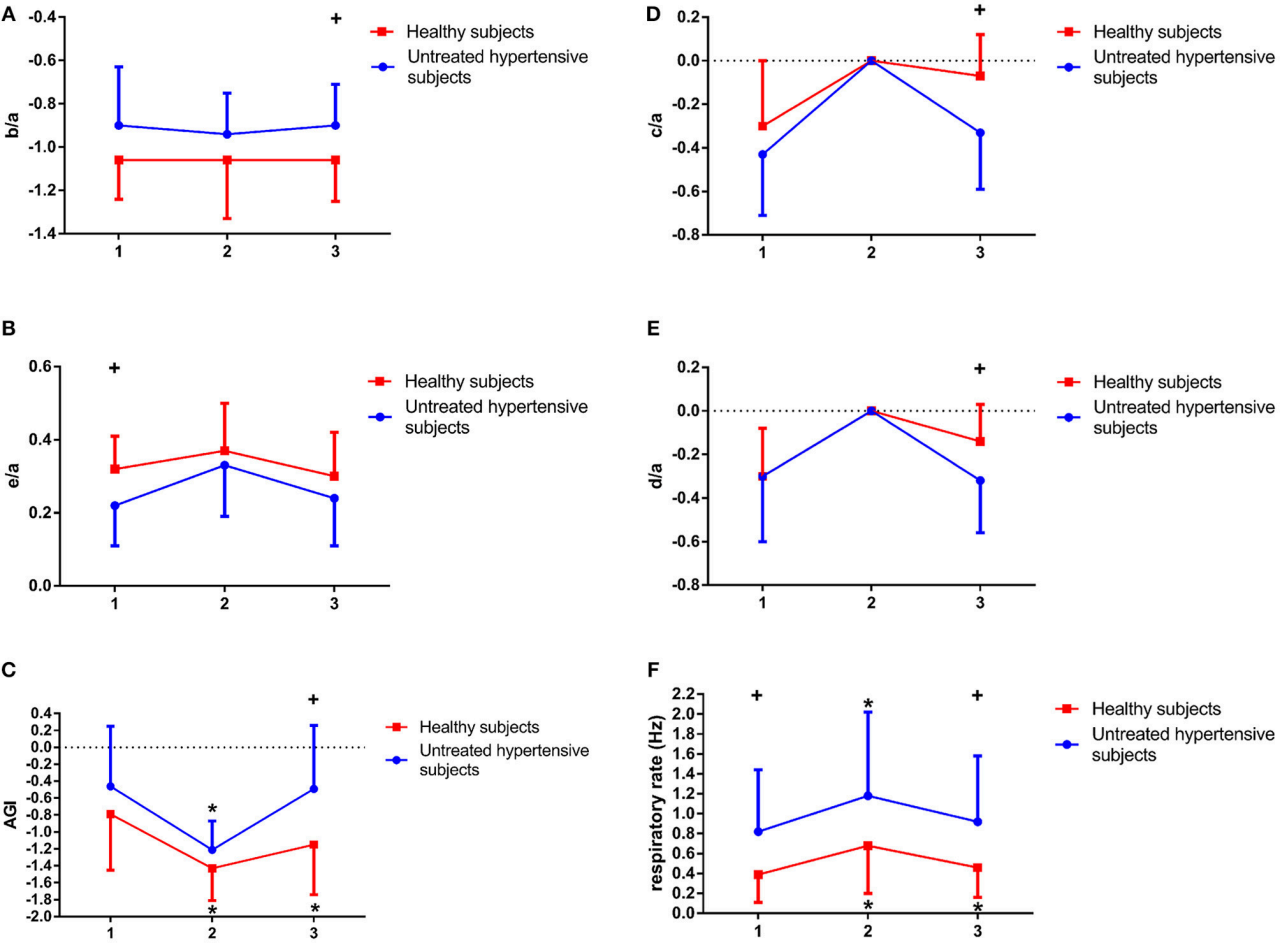
Differences of b/a **(A)**, e/a **(B)**, Aging index (AGI) **(C)**, c/a **(D)**, d/a **(E)**, and respiratory rate **(F)** before (1), during (2), and after exercise (3) in untreated hypertensive subjects (UHS) and healthy subjects (HS). ^+^Significant difference between UHS and HS: *P* < 0.05, * Significant difference from baseline within group: *P* < 0.05.

Before CPET, there were no significant differences between the two groups in b/a (−0.9 ± 0.27 vs. −1.06 ± 0.18, *p*-value = 0.114; Figure 8A), d/a (−0.3 ± 0.3 vs. −0.3 ± 0.22, *p*-value = 0.960; Figure 8E) and AGI (−0.46 ± 0.71 vs. −0.79 ± 0.66, *p*-value = 0.263; Figure 8C). However, after CPET, b/a (−0.9 ± 0.19 vs. −1.06 ± 0.19, *p*-value = 0.03) and AGI (−0.49 ± 0.75 vs. −1.15 ± 0.59, *p*-value = 0.011) were significantly higher in UHS than in the HS group. Whereas, d/a (−0.32 ± 0.24 vs. −0.14 ± 0.17, *p*-value = 0.024; Figure 8E) and c/a (−0.33 ± 0.26 vs. −0.07 ± 0.19, *p*-value = 0.004; Figure 8D) were significantly lower in UHS than in the HS. By contrast, before CPET, e/a (0.22 ± 0.11 vs. 0.32 ± 0.09, *p*-value = 0.007; Figure 8B) was significantly lower in UHS than that in HS, while there was no significant difference in this parameter (0.24 ± 0.13 vs. 0.3 ± 0.12, *p*-value = 0.146) after CPET between two the groups.

In addition, in comparison with before CPET, AGI was significantly increased during exercise in UHS (*p*-value = 0.003) and HS (*p*-value = 0.011), while after CPET AGI of UHS was significantly increased (*p*-value = 0.032) in Figure 8C. Moreover, during CPET, no differences were found between the two groups in c/a and d/a in Figures 8D,E.

### 3.2. Power spectral density analysis for respiratory rate

The changes in respiratory rate before, during and after CPET in the two groups are illustrated in Figure 8F. There was a significant difference in respiratory rate between UHS and HS before (0.82 ± 0.62 vs. 0.39 ± 0.28 Hz, *p*-value = 0.047) and after CPET (0.92 ± 0.66 vs. 0.46 ± 0.3 Hz, *p*-value = 0.046). However, there was no significant difference in respiratory rate during CPET (1.18 ± 0.84 vs. 0.68 ± 0.48 Hz, *p*-value = 0.095). In addition, respiratory rate in UHS was significantly higher during CPET (*p*-value = 0.000) and after CPET (*p*-value = 0.033) compared with before CPET. It was also significantly increased in HS during exercise in comparison with before CPET.

## 4. Discussion

This study demonstrated three interesting results. First, before CPET, there were no significant differences between the two groups in b/a, d/a, and AGI. However, after CPET, b/a and AGI were significantly higher in the UHS than in HS. Conversely, the d/a ratio was significantly lower in UHS than that in HS. Second, before CPET, e/a was significant lower in UHS than that in HS, while there was no significant difference in this parameter after CPET. Third, during CPET, AGI and respiratory rate were significantly increased in UHS and HS in comparison with before CPET.

The SDPPG is a simple, non-invasive, efficient, and inexpensive technique. We emphasize that there is no clear theoretical or experimental description of the link between these quantities and the underlying mechanical properties of the cardiovascular system. Nevertheless, the derived quantities reflect aspects of arterial stiffness and wave propagation and have been related to aging and to various cardiovascular pathologies. Thus, they provide a straightforward way of quantifying PPG signals and deriving useful prognostic and diagnostic information from them. SDPPG provides useful information in evaluating central and peripheral arterial properties to predict atherosclerosis or other cardiovascular risk factors (Mohanalakshmi and Sivasubramanian, 2012), especially for hypertension (Bortolotto et al., 2000). Moreover, respiratory function can also predict CVD, and there is a close relationship between vascular and respiratory function (Hjortdal et al., 2003; Barthel et al., 2013). Furthermore, in order to effectively evaluate and diagnose cardiovascular risk, it is clinically useful and perhaps essential to monitor cardiorespiratory function continuously and dynamically. Therefore, we aimed to investigate and compare the differences of cardiorespiratory function between UHS and HS, and to explore the potential causes for different cardiorespiratory exercise responses before, during and after CPET. To the best of our knowledge, this is the first report to investigate and compare cardiorespiratory function in UHS and HS before, during and after exercise by using SDPPG analysis.

In this study, there were no significant differences in b/a, d/a, and AGI between the two groups before CPET. However, after CPET, there were significant differences in these parameters between UHS and HS. These interesting observations show that exercise can be an effective way to investigate differences in cardiovascular function between healthy and UHSs. It has been shown that b/a is significantly decreased with atherosclerosis (Imanaga et al., 1998). It has also been reported that b/a reflects the distensibility of large arteries (Rivas-Vilchis et al., 2007), and the difference of distensibility of large arteries also suggested the different response of systolic blood pressure in hypertension. Elevated SBP was associated with increase of arterial stiffness (Otsuka et al., 2007; Yousef et al., 2012). The d/a ratio has been associated with augmentation index, which is a predictor of CV events, such as target organ damage (Shimizu and Kario, 2008; Tomoyuki and Toshiaki, 2013). Augmentation index is also related to central blood pressure (Munir et al., 2008). In other words, d/a is associated with augmentation of blood pressure in the aorta by wave reflection, which in turn, is an indirect index of arterial stiffness (Tomoyuki and Toshiaki, 2013). The parameter AGI has been used to assess the vascular aging and for screening of arteriosclerotic disease (Rivas-Vilchis et al., 2007). In addition, the elevated AGI was associated with blood pressure, obesity and smoking, which can lead to higher aortic stiffness and can be even in the absence of atherosclerosis of the blood vessel (Mahmud and Feely, 2003; Tabara et al., 2008). In this study, the smoking history of UHS was a little higher than that of HS, although there was no significant difference in smoking history between two groups. The relations between AGI and smoking or other risk factors need to be further explored.

Some studies based on large samples have found that a lower b/a, a higher d/a, and a higher AGI indicated increased arterial stiffness and were a marker of arteriosclerosis (Hashimoto et al., 2002, 2005). Moreover, the changes of these three parameters were good markers of the effects on vascular pathophysiology evaluating the arterial distensibility of large and peripheral arteries in hypertension (Rivas-Vilchis et al., 2007). In contrast, others have reported that these three parameters exhibited limited ability to detect pathophysiological changes in large arteries in patients with end-organ damage (Tabara et al., 2016). It was further suggested that SDPPG indices might be more closely related to pathophysiological changes in small vessels than in large arteries (Tabara et al., 2016). However, our findings support are in line with the consensus mentioned above and suggest that SDPPG can reveal the differences of vascular function in CVD, especially when combined with exercise. The present study also showed that there was a significant difference in the e/a ratio between the two groups before exercise. However, after exercise there was no significant difference in this parameter. Takazawa et al. demonstrated that lower e/a reflected increased arterial stiffness (Takazawa et al., 1998). However, others have suggested that the late systolic indices (d/a and e/a), which are predominantly associated with pressure augmentation, did not provide independent information about vascular function (Simek et al., 2005). In this study, we observed that e/a was significantly lower in UHS than that in HS, suggesting that a difference in e/a between the two groups during CPET may predict the potential risks of increased arterial stiffness.

During exercise, both c/a and d/a were not detectable in either group. It has been reported that a reduced c/a was associated with a decreased baroreflex responses and with a reduced cardiac parasympathetic tone (Kohjitani et al., 2014). Moreover, the c/a was related to hypertension, diabetes mellitus and hyperlipidemia as well as elevated total cholesterol concentrations (Kohjitani et al., 2014). In addition, some studies have reported that a decline in these parameters was associated with increased arterial stiffness or age (Rivas-Vilchis et al., 2007; Rozi et al., 2013). However, the pathophysiological significance of disappearance of c/a and d/a was still unclear. In this study, some features of the PPG waveforms disappeared during exercise. It has been reported that the diastolic peak of the PPG moved backward with an increase of exercise load, and gradually returned to baseline after exercise (Wang et al., 2017). The disappearance of the c and d points may be related to the different exercise intensities or poor data quality during exercise. There were no c and d points in two of the UHS before exercise, while in these two subjects the c and d points appeared after exercise. This phenomenon may be influenced by vascular response. It should be further explored in detail in the future. In this study, the significant differences of respiratory rate were found between two groups before and after CPET. There was a significant difference in this variable during exercise compared with before exercise. A study found that it predicted cardiovascular disturbance, ventilatory control and pulmonary perfusion after acute myocardial infarction (Barthel et al., 2013). The change of respiratory rate was also a reflection of cardiac autonomic control. The higher respiratory rate in UHS reflected the disturbed autonomic control to some extent (Barthel et al., 2013; Rozi et al., 2013). It has been found that the autonomic nervous system plays key roles in modulating cardiovascular function and in controlling BP, both at rest and in response to environmental stimuli (Mancia and Grassi, 2014). Moreover, sympathetic nerve activity, arterial baroreflex sensitivity and cardiac vagal drive are the main regulators of BP in response to interventions, such as exercise (Grassi et al., 1994, 1998; Carter et al., 2003).

Furthermore, respiratory function is closely coupled to vascular function. A study reported that muscular sympathetic nerve activity significantly increased in response to exercise, together with significant increases in HR, respiratory rate and skin blood flow (Brown et al., 2013; White and Raven, 2014). Additionally, it has been shown that respiratory rate is associated with blood flow during exercise (Hjortdal et al., 2003). The faster breathing and longer inspiratory phase were associated with pulmonary flow during exercise. Exercise promoted cardiac pump function, which increased cardiac output (CO), predominantly by raising the HR and only to a lesser extent by raising stroke volume (Pedersen et al., 2002). In this study, the incremental exercise increased peripheral muscle contraction which promoted an immediate increase in venous return and thus, CO. Moreover, during exercise, an enhancement in ventilator response promoted a cardiovascular response, which increased the demand for oxygen and accelerated blood flow and CO (Turner, 1991; Ramponi et al., 2013). This activation led to shear stress-mediated augmentation in endothelial cell NO production resulting in caused vasodilatation (Horiuchi and Okita, 2012). Therefore, interaction of multiple physiological signals contributed to the differences in cardiorespiratory function between the two groups.

Some potential limitations of the present study should be emphasized. Firstly, the number of subjects investigated was relatively small. Secondly, there were significant differences in BMI and weight between two groups because most of hypertensive subjects were a little overweight. Thirdly, biochemical parameters, such as blood lipids, have not been measured in the present study, which might have provided some additional underlying mechanisms for the different responses during and after CPET.

## 5. Conclusion

Differences in the response to exercise revealed differences in cardiorespiratory function between UHS and HS. These differences not only highlight the CV risk of UHS, but also can predict the development of increased arterial stiffness in these subjects. Additionally, differences in AGI, autonomic nervous function and respiratory activity were found between the two groups during CPET in comparison with before CPET. In summary, this study demonstrated that the response to exercise is an effective way to characterize differences in cardiorespiratory function between untreated hypertensives and healthy controls and to provide useful predictive and diagnostic information for CVD.

## Author contributions

YZ, XS, and LX proposed the scientific problems. YZ, XS, LQ, and LX designed the experiments. YZ, XC, YL, and TZ collected the experimental data. YZ and ZJ processed and calculated the data. YZ conducted statistical analysis and wrote the draft manuscript. SG, LX, XS, and LQ contributed to the revision and final version of manuscript.

### Conflict of interest statement

The authors declare that the research was conducted in the absence of any commercial or financial relationships that could be construed as a potential conflict of interest. The handling editor declared a past co-authorship with one of the authors LX.
